# A calibration method for smartphone camera photophlethysmography

**DOI:** 10.3389/fdgth.2023.1301019

**Published:** 2023-11-24

**Authors:** Yinan Xuan, Colin Barry, Nick Antipa, Edward Jay Wang

**Affiliations:** ^1^ Electrical and Computer Engineering, UC San Diego, La Jolla, CA, United States; ^2^ The Design Lab, UC San Diego, La Jolla, CA, USA

**Keywords:** smartphone, photoplethysmography, calibration, pulse oximetry, hemoglobin, camera

## Abstract

Smartphone camera photoplethysmography (cPPG) enables non-invasive pulse oximetry and hemoglobin concentration measurements. However, the aesthetic-driven non-linearity in default image capture and preprocessing pipelines poses challenges for consistency and transferability of cPPG across devices. This work identifies two key parameters—tone mapping and sensor threshold—that significantly impact cPPG measurements. We propose a novel calibration method to linearize camera measurements, thus enhancing consistency and transferability of cPPG across devices. A benchtop calibration system is also presented, leveraging a microcontroller and LED setup to characterize these parameters for each phone model. Our validation studies demonstrate that, with appropriate calibration and camera settings, cPPG applications can achieve 74% higher accuracy than with default settings. Moreover, our calibration method proves effective across different smartphone models (N=4), and calibrations performed on one phone can be applied to other smartphones of the same model (N=6), enhancing consistency and scalability of cPPG applications.

## 1. Introduction

Photoplethysmography (PPG) is a widely used measurement technique in the medical field that involves illuminating a vascularized tissue and tracking the reflected light over time. This pivotal measurement is adept at capturing an array of physiologically meaningful information, such as heart rate (1–5), heart rate variability (6, 7), blood oxygenation (5, 8, 9), hemoglobin concentration (10, 11), vascular aging (12), blood pressure (5, 13–15), diabetes (16), and respiratory rate (17). A PPG measurement is realized with a light source and a photo sensor pair. This combination is ubiquitous in today’s smartphone devices, which employ software-accessible cameras, flash LEDs, and screens. By placing a person’s finger over the light source and sensor, the variation in blood volume in the finger during a cardiac cycle leads to maximal absorption of light at the peak cardiac output (systole) and minimal absorption at the trough (diastole).

This paper refers to the use of smartphone cameras to measure PPG as Camera PPG (**cPPG**). Despite a robust body of research surrounding cPPG, no systematic investigation has been undertaken to ascertain the reliability of the cPPG technique in creating accurate measurements under different camera parameter settings and across smartphone devices. The effects of camera parameters and differing components of various smartphone devices may interfere with the consistency of PPG measurements across devices. This paper proposes a methodology for setting smartphone camera parameters and calibration methods that reliably record PPG amplitudes critical for measurements such as hemoglobin concentration and blood oxygenation calculations.

Understanding the results presented in this paper requires insight into how smartphone cameras rely on modern computational camera systems. These systems incorporate a multitude of image quality-enhancing techniques designed to replicate the human vision system. However, human vision is non-linear (18). Specifically, humans do not perceive brightness linearly. There is an innate adjustment of visual dynamic range, which enhances dark areas and suppresses bright areas, thereby making an entire scene appear visible. This effect is implemented in computational cameras through a process called tone mapping (19), commonly in the form of a logarithmic function for typical color spaces such as sRGB.

In the context of PPG, the most crucial measurement is the comparison between the baseline amplitude, commonly referred to as DC, and the pulse amplitude, often referred to as AC. It’s the ratio of AC against DC across different color channels that offers insights into the blood’s absorption properties, a measurement known as the Ratio-of-Ratios (RoR). Errors in absorption measurements are detrimental to blood oxygenation and hemoglobin concentration measurements. However, tone mapping distorts the DC and AC amplitudes, grossly overestimating the DC component by amplifying low-amplitude signals and underestimating the AC component by attenuating high-amplitude signals. Additionally, the function used in tone mapping is dynamically adjusted depending on the scene, leading to amplitude inconsistencies across scenes depending on the underlying tone map. This major issue remains unaddressed in previous cPPG research, likely leading to inaccurate cPPG measurements across a broad range of applications.

Our background investigation revealed that only a few approaches have considered controlling the multitude of camera parameters that exist in modern smartphones. Most prior works largely depended on post-processing after allowing the complex camera system to automatically capture a video, with auto-exposure, auto-white balancing, and video compression algorithms altering the raw signal in non-linear ways (4, 6, 20–28). Only a handful of works have addressed changing exposure by disabling auto-exposure (29–31); calculating absorption frame-by-frame in an online manner to avoid compression; attempting to control white balance by designating a preset, most commonly an incandescent setting to emphasize blue and green channels (32–34); or opting to control each color channels with individual gain settings (8, 11). Although these works hint at the right direction in addressing the issues, our investigation finds that these solutions are inadequate, and in fact, overlook some of the most crucial camera parameters affecting cPPG measurements.

In order to address the nonlinearities, prior works heavily rely on using data-driven models. Such solutions usually involve measuring the target biomarker (i.e. pulse oximetry, hemoglobin concentration) on one phone and a reference clinical device. The performance of such data-driven solutions is based on a strong assumption that the nonlinear preprocessing will be (1) the same under new conditions such as a different user with darker skin tones, (2) the same under updates to camera algorithms, and (3) the same on different phones. However, none of these are guaranteed.

**Figure 1:**
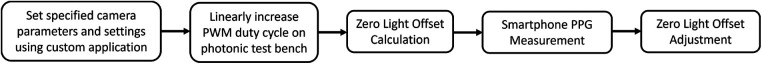
Block diagram of smartphone calibration procedure for reliable PPG measurements.

In this paper, we propose a guide to proper camera settings and a scalable calibration method for reliable smartphone cPPG measurements. The proposed calibration method relies on a photonic test bench design that employs a light-blocking box and off-the-shelf LEDs. The device outputs a range of LED brightnesses in RGB using Pulse-Width Modulation (PWM) to emulate a variety of PPG amplitudes, thereby mimicking different optical signals resulting from a range of blood compositions. Complementing the calibration device is a custom Android application that uses the Camera2 API to measure the calibration input and calculate the smartphone model-specific sensor characteristic, which involves the measurement of the minimum light threshold. This minimum light threshold value, referred to as the zero light offset (ZLO) in this paper, is crucial for correcting the amplitude measured by the camera, as it directly affects the DC component of the PPG measurement. The overall calibration procedure is outlined in a block diagram in Figure 1 Without this correction, the ratiometric calculation would grossly overestimate the effect of the AC measurement.

We evaluate the performance of the proposed calibration method through a series of simulation studies. These studies use the test bench device to emulate the optical signal of different ratios of AC and DC intensities across color channels, thus producing a range of Ratio of Ratios (RoR) from 0.5–2.0. This range represents the expected operational range of typical pulse oximeters. To assess the effectiveness of our calibration method, we compare the RoR calculated from the signal captured using three settings: Default Auto Tone Mapping, Uncalibrated Linear Tone Mapping, and Calibrated Linear Tone Mapping. We find that by properly setting the tone mapping to a linear mode and calibrating the sensor for its ZLO for each phone model (N=4), the calibrated results exhibit 74% and 60% lower Mean Absolute Error (MAE) compared to the Default automatic setting and the uncalibrated linear setting, respectively. Similarly, the $R^{2}$ is improved to 0.97 from 0.81 and 0.72. Furthermore, we find that the calibration found for one phone of a model can be used for other phones of the same model without recalibration (N=6). These findings strongly advocate for the importance of our calibration method in reliably measuring PPG amplitude to faithfully reproduce RoR values that are needed to measure optical blood absorption.

### 1.1. Smartphone camera PPG (cPPG)

Smartphone cameras, extensively explored as sensors for pulse measurements at the finger via photoplethysmography (PPG) (3), utilize a common method where the finger covers either the LED or the screen, referred to as Camera PPG (cPPG). This method contrasts with Remote PPG (rPPG) (35), which captures the PPG signal non-contactly by detecting absorption fluctuation at a distance. While this paper primarily focuses on cPPG due to its widespread study and public use, the introduced findings and techniques remain pertinent to rPPG measurements and invite future exploration.

Various digital biomarkers, including heart rate (4, 20–24, 36, 37), heart rate variability (6, 22, 24–26), and breathing rate (27, 28, 38, 39), can be estimated through cPPG. These estimations are largely frequency-dependent, thus remaining unimpacted by calibration absence since signal amplitude and color channel relationships, rather than frequency information, are primarily influenced by camera alterations. Conversely, amplitude-dependent measurements like pulse oximetry (5, 30, 40–42) and hemoglobin concentration (31, 43–45), are significantly affected by a lack of calibration, thereby rendering them unreliable due to sensing variations from the complex smartphone camera system.

**Figure 2:**
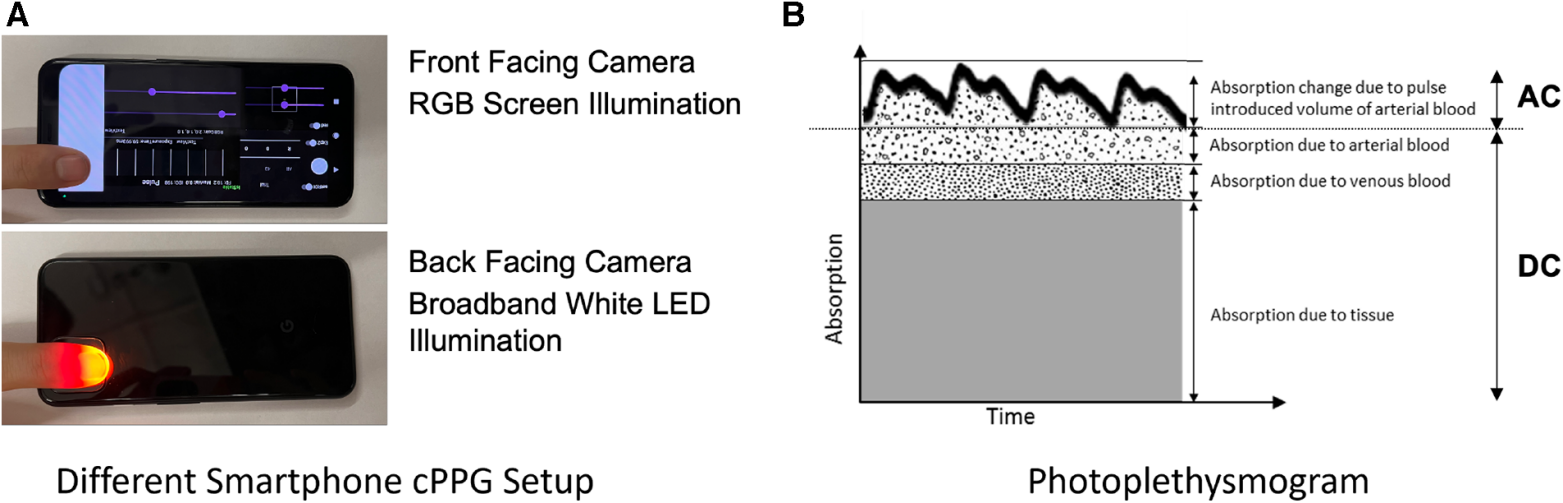
(**A**) Two common forms of smartphone PPG using the front camera with the screen and the back camera with the flash LED (**B**) Light absorbed by living tissue. Adapted from Webster et al. (47). The absorption of light changes due to the change in the volume of blood when the heart pulses.

Blood composition measurements are based on calculating the ratio of amplitudes between different PPG wavelengths, capturing the baseline(DC) and the fluctuation (AC) of absorption. As depicted in Figure 2B, the AC component arises from blood volume changes as the heart pumps blood with each beat. The goal of any blood color analysis is to measure the ratio of AC/(AC+DC) in the signals between two color channels, capturing blood’s absorption at specific wavelengths (46). Ensuring accurate measurement of this RoR is vital for blood color and blood composition analysis. This paper identifies how standard smartphone camera applications with automatic tone mapping settings introduce a nonlinear scaling of the measurement due to dynamic range adjustments, leading to significant inaccuracies in DC and AC measurements and thus affecting the RoR calculation and blood color and blood composition analysis accuracy. To rectify this issue, a method is proposed to correct these errors and obtain accurate measurements, enabling more reliable blood color and composition analysis using smartphone-based PPG systems.

### 1.2. Smartphone computational camera complexity and control

Despite cPPG’s attempt to utilize smartphone cameras as photosensors, the camera is far more complex, undergoing various hardware and software manipulations. This paper primarily focuses on the control developers have over these camera characteristics via Application Programming Interfaces (APIs). If certain control over the camera is not exposed at the API level, an app cannot exert that control. While such limitations can be circumvented if a smartphone is rooted, allowing custom flashing of modified firmware, this solution is often blocked and not user-friendly, making it unsuited for the app ecosystem.

#### 1.2.1. Access and compensations of smartphone configurations for cPPG

Because prior works did not fully control the nonlinear effects introduced by nonlinear tone mapping, the resulting PPG measurement incorporates such nonlinearities. However, prior works have still demonstrated the feasibility of smartphone cPPG for the many applications we have mentioned here. This can largely be credited to the use of data-driven models.

Several works have used data-driven models to successfully measure blood oxygenation using the smartphone camera with the flash LED for SpO2 measurement, achieving an MAE of 5% or less (8, 9). For anemia screening via HgB measurement, Wang et al. (10) leveraged a Support Vector Machine (SVM) to train a model utilizing various combinations of amplitude and ratios achieving a rank order correlation of 0.82. In a later study, they fitted a linear regression model (11). Other approaches in the field use artificial neural networks (43), a variety of machine learning methods (31), or linear regression with data collected from an array of LEDs (44).

Notably, smartphone cPPG-based solutions for SpO2 measurements have depended on data-driven techniques, even though a simple ratio between red and green absorptions should theoretically suffice. This reliance on complex models may be due to the need to reverse the nonlinearities imposed by the camera and learn the relationship between the PPG and the target measurement. However, relying on data-driven models to simultaneously address both issues has multiple implications. First, the nonlinearities can differ from phone to phone, and no work clearly demonstrates that a system trained on one phone can be deployed on another, potentially necessitating device-specific training and a new dataset for the algorithm to work on a new phone. Secondly, nonlinearities can vary depending on measurement conditions as the tone mapping curve may change if not under control. The proposed solution in this paper linearizes the measured signal, allowing for directly comparable PPGs from phone to phone and from one exposure setting to another.

The remainder of the paper is organized in order as follows: (2) the methods section detailing the proposed settings, calibrations procedure, and evaluation process; (3) the results section presenting data from the evaluation; (4) the discussion section commenting on the importance, implications, and limitations of the proposed work; and (5) the conclusion providing the final thoughts for the reader.

## 2. Methods

### 2.1. Benchtop calibration device

**Figure 3:**
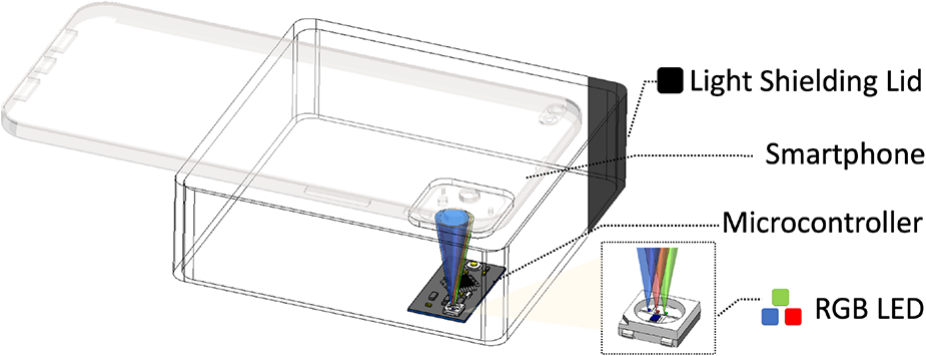
Bench top calibration set up.

A test bench device was designed utilizing commodity off-the-shelf components, as depicted in Figure 3. The primary elements of the test bench include a black 3D printed light shield that connects a smartphone camera to a dark chamber, and a microcontroller (specifically, a Qduino development board) situated within the dark chamber that illuminates the camera scene with a pulse width modulation (PWM) controllable RGB LED.

The PWM frequency was empirically determined to be 32 MHz to avoid the rolling shutter effect. The primary objective of the test bench is to establish a controlled environment, enabling us to emulate various PPG measurement conditions in reproducible experiments where the AC and DC inputs are known and adjustable. This test bench setup also provides a platform to perform a calibration technique that we have identified as necessary for each phone to compute the RoR accurately.

### 2.2. Smartphone settings

Paired with the calibration device is a custom Android application that leverages the Android Camera2 API for comprehensive control over the camera’s parameters. The proposed calibration method relies on this API to reliably yield accurate cPPG measurements. An important aspect of the smartphone setup is the intentional avoidance of many default automatic “enhancements.” Configurations accessible via the Camera2 API, such as manually setting and locking the white balance, sensor exposure time, frame rate, and sensitivity boost, are manipulated for this purpose. These parameters may be modified before or after the measurements, but need to remain consistent during a cPPG measurement. Furthermore, to bypass the automatic image adjustments typical of smartphone camera systems, the image processing pipeline must be tuned to produce reliable images for measurements. This involves specifying the color correction settings, including the color correction mode, gain, and transformation matrix.

#### 2.2.1. Tone mapping

Tone mapping is a key parameter for linear and reproducible cPPG measurements that has not been previously explored. The default setting for tone mapping in Android is called FAST and is nonlinear. In this paper, we refer to it as Default Automatic. Default Automatic adapts the control points of the previously used tone map based on the scene. The adaptive control causes nonlinear effects that cannot be reversed in post processing. For proper calibration, the tone mapping parameter should be set to linear by setting the tone mapping mode to *CONTRAST_CURVE* and manually setting two control points as *[(0, 0), (1.0, 1.0)]* for all color channels in the tone curve parameter.

**Figure 4:**
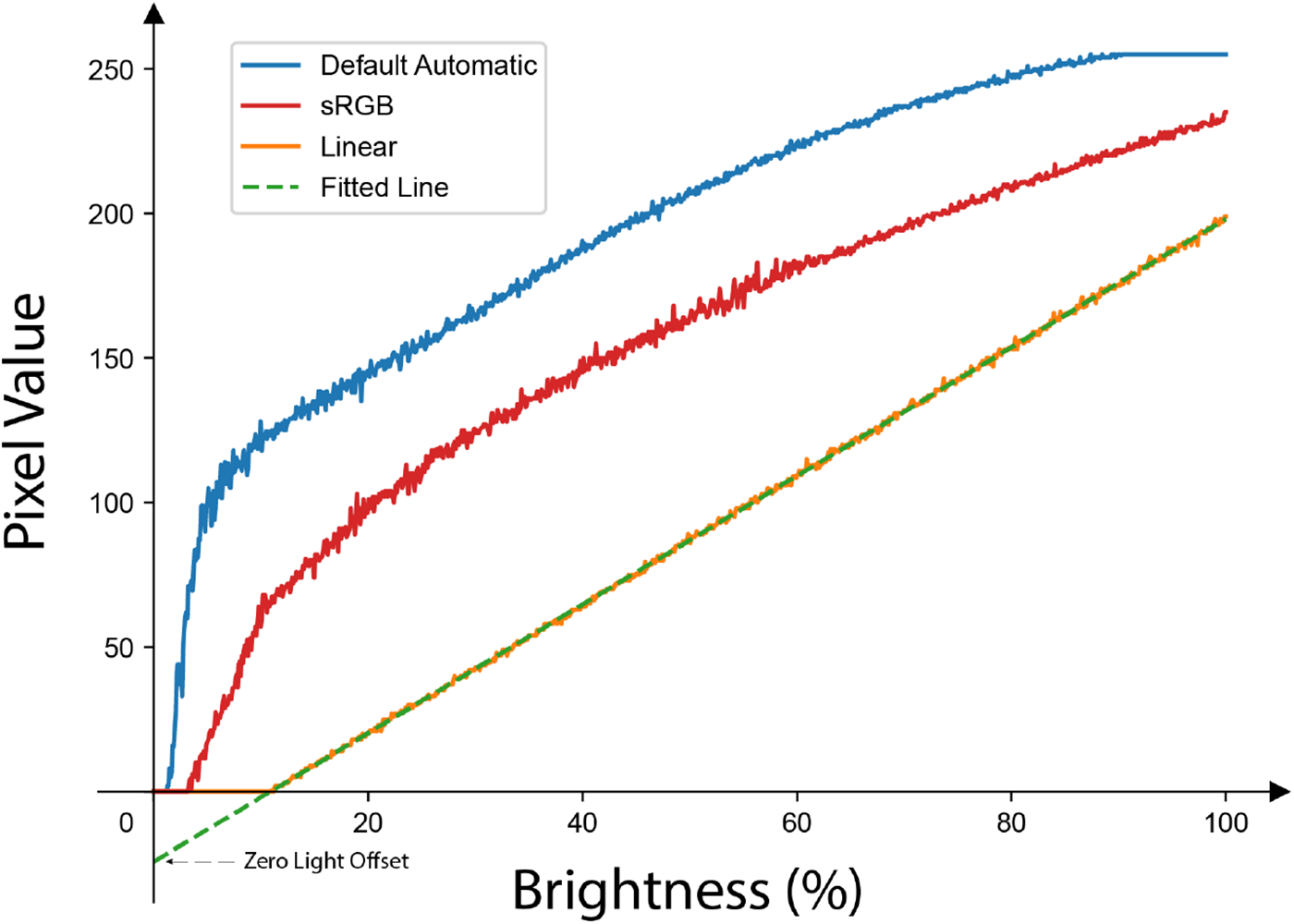
The response of the same pixel to a linearly increasing amount of brightness under different tone mapping settings: only the Linear tone mapping setting preserves the linearity of the original signal. Note the y-intercept (indicated as Zero Light Offset in this paper) of the fitted line is not zero and it is different for different smartphone models as shown in Table 1.

For this paper, the Default Automatic tone mapping parameter serves as a default for comparison against the current state of the art employed in many prior works. Earlier cPPG studies typically used the default setting, and, to our knowledge, most camera applications use sRGB or other nonlinear tone maps. Figure 4 illustrates the impact of 3 different tone mapping settings across a range of incident light intensities: Linear, sRGB, and Default Automatic. It is evident that the linear measurement exhibits a high degree of linearity, which indicates that the camera’s CMOS sensor is well-engineered for a linear response. Meanwhile, a nonlinear tone mapping significantly alters the signal, rendering the measured pixel values unsuitable for cPPG measurements or RoR calculations.

**Table 1:** ZLO characterized for different phone models.

Phone model	ZLO
Pixel 4	−22.5
Pixel 7	−14
Samsung S22	−19.6
Motorola G 2022	−14.9

#### 2.2.2. Zero light offset

As illustrated in Figure 4, a fitted line of brightness-to-pixel value via linear tone mapping results in a non-zero y-intercept, which we refer to as the ZLO. This phenomenon is due to the intrinsic detection threshold of the camera’s photosensor, with different smartphone models exhibiting varying hardware or firmware settings that influence the camera sensor.

The presence of a negative y-intercept ZLO implies that an increase in the measured pixel value does not proportionally reflect the actual increase in brightness, thus inducing errors in RoR calculations. It is essential to recognize that while the slope of this line affects all measurements uniformly and hence cancels out in the RoR calculations, the ZLO manifests as an additive shift. This shift can distort the relative differences between the two wavelengths, especially when the pixel values are low, thereby affecting the final RoR.

### 2.3. Calibration procedure

For accurate and reliable cPPG measurements with a specific phone model, it is essential to calibrate of ZLO, which is a critical hardware-specific parameter. As discussed in Section 2.2.2, ZLO significantly influences the computation of RoR and other proportionality metrics, all of which are fundamental in determining hemoglobin concentration or pulse oximetry. Therefore, the calibration process must carefully account for ZLO within the constraints of the recommended camera settings.

The calibration process requires the gradual escalation of LED brightness on the test bench by linearly increasing the PWM duty cycle, while a single pixel at a fixed position on the smartphone records the intensity. It is important to ensure that the smartphone camera is configured with the aforementioned settings, which include linear tone mapping. The pixel values recorded can then be fitted into a simple line to characterize the ZLO. Characterizations on N=4 phone models confirmed that different phone models indeed have distinct ZLO values (Pixel 4: −22.5; Pixel 7: −14; Galaxy S22: −19.6; Moto G 2022: −14.9) (Table 1).

To apply the calibration, this ZLO can serve as a correction in RoR calculations to avoid additive shifts. By subtracting the ZLO from the recorded pixel value, we can normalize the pixel values, thereby yielding an accurate RoR. This adjustment for the ZLO ensures that the intrinsic camera detection threshold does not skew the final RoR measurements.

### 2.4. Evaluation

**Figure 5:**
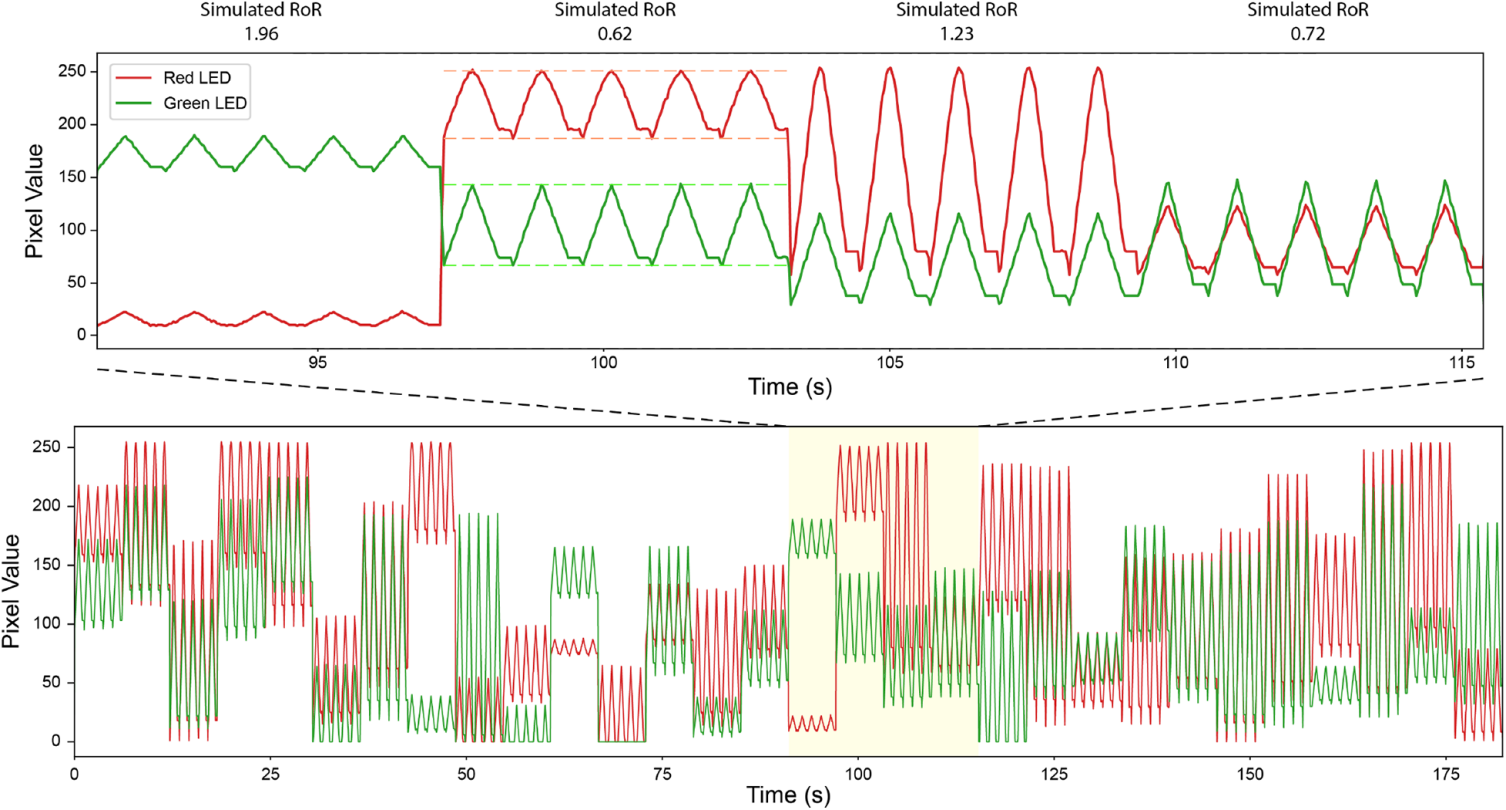
Simulated Signal to Create Pulses w/Controlled RoR. Bottom: a portion of simulated signals. Top: a zoomed in version. Dashed line indicates peak and troughs. The data in the plot are recorded with Pixel 4 under Linear tone mapping setting.

An evaluation process was designed to assess the reliability of the ZLO correction in accurately retrieving the RoR value. We created a simulation with signals that produced a range of RoRs, using the red and green LEDs from the test bench to emit controlled brightness levels. The validation process involves sweeping through a variety of DC and AC value combinations for both the red and green LEDs to randomly sample RoRs between 0.5 and 2 for 100 iterations (Figure 5). Each combination was repeated five times, with a 100 millisecond gap between each repetition. Prior to the simulation, the duty cycles of both LEDs were set to 100%, and two pixels were selected, one for each color channel, with a reading close to 250 in the corresponding color channel. This ensured that neither pixel would be saturated during the simulation, while maintaining as much dynamic range as possible. For each smartphone, the same set of signals were recorded twice using Default Automatic and Linear tone mapping settings.

Our study utilized nine phones: Google Pixel 4 (N=6), Google Pixel 7 (N=1), Samsung Galaxy S22 (N=1), and Motorola Moto G 2022 (N=1). For the camera settings, auto-exposure was disabled and an exposure time of 30 ms, a sensitivity of 55, and a sensitivity boost of 100 were employed. Auto-white balancing was also turned off, with the color correction gains set to 2 for red and blue, and 1 for both green_even and green_odd. An identity matrix was used for the color transform matrix. Pixel values were extracted per frame in real-time to avoid artifacts from the video compression algorithm. When recording for an LED of a specific color, a pixel was selected where there was no overlap with any neighboring LED of a different color.

During data analysis, the recorded signals were divided into 100 segments of equal length, each containing five simulated pulse repetitions. The first and last pulse were discarded during data cleaning, and the maximum and minimum values were identified as the peak and the trough for the simulated pulse, respectively. This approach mitigates potential inaccuracies due to variations in sampling rate. The AC was then defined as the difference between the peak and the trough, and the DC as the value of the trough. If the DC of any color channel was 0, the segment was discarded. Finally, 50 segments were randomly selected for RoR calculation. The RoR values were calculated based on Eq. 1, where $\lambda_{1}$ is red and $\lambda_{2}$ is green. For recordings with the Linear tone map, in addition to RoR calculated from raw pixel values (Uncalibrated Linear) (Eq. 1), RoR was also calculated with the calibration taken into account (Calibrated Linear) (Eq. 2). The results from these studies are orgnized into regresion plots, bland altman plots, and tables in the results section.

(1) $$\text{Uncalibrated \textbackslash{} Ratio \textbackslash{} of \textbackslash{} Ratios}=\frac{{\mathrm{AC}}_{\lambda 1}/({\mathrm{AC}}_{\lambda 1}+{\mathrm{DC}}_{\lambda 1})}{{\mathrm{AC}}_{\lambda 2}/({\mathrm{AC}}_{\lambda 2}+{\mathrm{DC}}_{\lambda 2})}$$

(2) $$\text{Calibrated \textbackslash{} Ratio \textbackslash{} of \textbackslash{} Ratios}=\frac{{\mathrm{AC}}_{\lambda 1}/({\mathrm{AC}}_{\lambda 1}+{\mathrm{DC}}_{\lambda 1}-\mathrm{ZLO})}{{\mathrm{AC}}_{\lambda 2}/({\mathrm{AC}}_{\lambda 2}+{\mathrm{DC}}_{\lambda 2}-\mathrm{ZLO})}$$

## 3. Results

### 3.1. Across phone model validation

**Figure 6:**
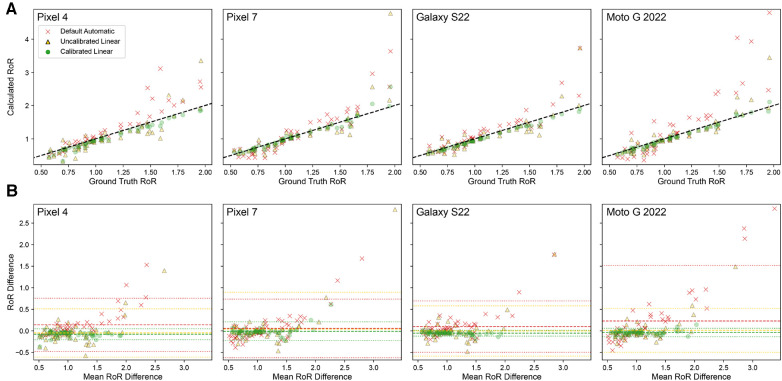
Using calibration in combination with the Linear tone mapping setting significantly improves RoR accuracy for all four phones of different models. (**A**) Correlation plot of ground truth RoR VS Calculated RoR. RoR calculated with Calibrated Linear has the lowest MAE and is highly correlated to the ground truth. The dashed line indicates y=x. (**B**) Bland-Altman Plot. The dashed line indicates Bias; the dotted lines indicate limits of agreement (LoA).

Figure 6 presents calculated RoR values from different tone mapping settings for four different phone models: Pixel 4, Pixel 7, Galaxy S22, and Moto G 2022. As observed, the Calibrated Linear RoRs demonstrates a smaller absolute error compared to both the Uncalibrated Linear RoRs and the Default Automatic RoRs across all models. Specifically, for Pixel 4, the Calibrated Linear RoRs’ absolute error is 58% less than that of the Uncalibrated Linear RoRs and 56% less than that of the Default Automatic RoRs, as determined by paired t-tests with p-values of 0.009 and 0.0014, respectively. Similarly, for Pixel 7, the absolute error reductions for the Calibrated Linear RoRs compared to the Uncalibrated Linear RoRs and Default Automatic RoRs are 72% ($p\text{-value}=3.2\times 10^{-6}$) and 64% (p-value=0.031), respectively. For Galaxy S22, the reductions are 66% ($p\text{-value}=8.6\times 10^{-3}$) and 61% (p-value=0.026), and for Moto G 2022, they are 87% ($p\text{-value}=2.2\times 10^{-4}$) and 62% (p-value=0.011). Across devices, on average, the reductions are 74% (p-value=0.01) and 60% ($p\text{-value}=5\times 10^{-4}$) (Table 2).

**Table 2:** Statistical analysis of RoR calculations for different tone mapping settings across four phone models.

		Device	Pixel 4	Pixel 7	Galaxy S22	Moto G 2022	Mean	STD
Correlation	MAE	Default automatic	0.19	0.21	0.16	0.38	0.24	0.10
		Uncalibrated linear	0.18	0.16	0.14	0.13	0.15	0.02
		Calibrated linear	0.079	0.058	0.054	0.05	0.06	0.01
	$R^{2}$	Default automatic	0.86	0.84	0.78	0.77	0.81	0.04
		Uncalibrated linear	0.73	0.63	0.7	0.8	0.72	0.07
		Calibrated linear	0.97	0.93	0.99	0.98	0.97	0.03
Bland-Altman	Bias	Default automatic	0.14	0.055	0.23	0.099	0.13	0.07
		Uncalibrated linear	−0.049	0.041	0.013	−0.0022	0.00	0.04
		Calibrated linear	−0.078	−0.0093	−0.037	−0.053	−0.04	0.03
	LoA lower	Default automatic	−0.48	−0.63	−1.1	−0.5	−0.68	0.29
		Uncalibrated linear	−0.61	−0.81	−0.5	−0.59	−0.63	0.13
		Calibrated linear	−0.2	−0.23	−0.14	−0.12	-0.17	0.05
	LoA upper	Default automatic	0.76	0.74	1.5	0.69	0.92	0.39
		Uncalibrated linear	0.51	0.89	0.52	0.58	0.63	0.18
		Calibrated linear	0.047	0.21	0.061	0.015	0.08	0.09

The table includes Mean Absolute Error (MAE), $R^{2}$ values for correlation analysis, Bias, and Limits of Agreement (LoA) for Bland-Altman analysis for Default Automatic, Uncalibrated Linear, and Calibrated Linear settings. Mean and standard deviation (STD) values are also included.

The performance of each smartphone with respect to the RoR of different settings are visualized with the regression and bland alman plots in Figure 6. These paired t-test results corroborate that for all tested phone models, using calibration in combination with the Linear tone mapping setting significantly improves the accuracy of RoR calculations.

### 3.2. Within phone model validation

**Figure 7:**
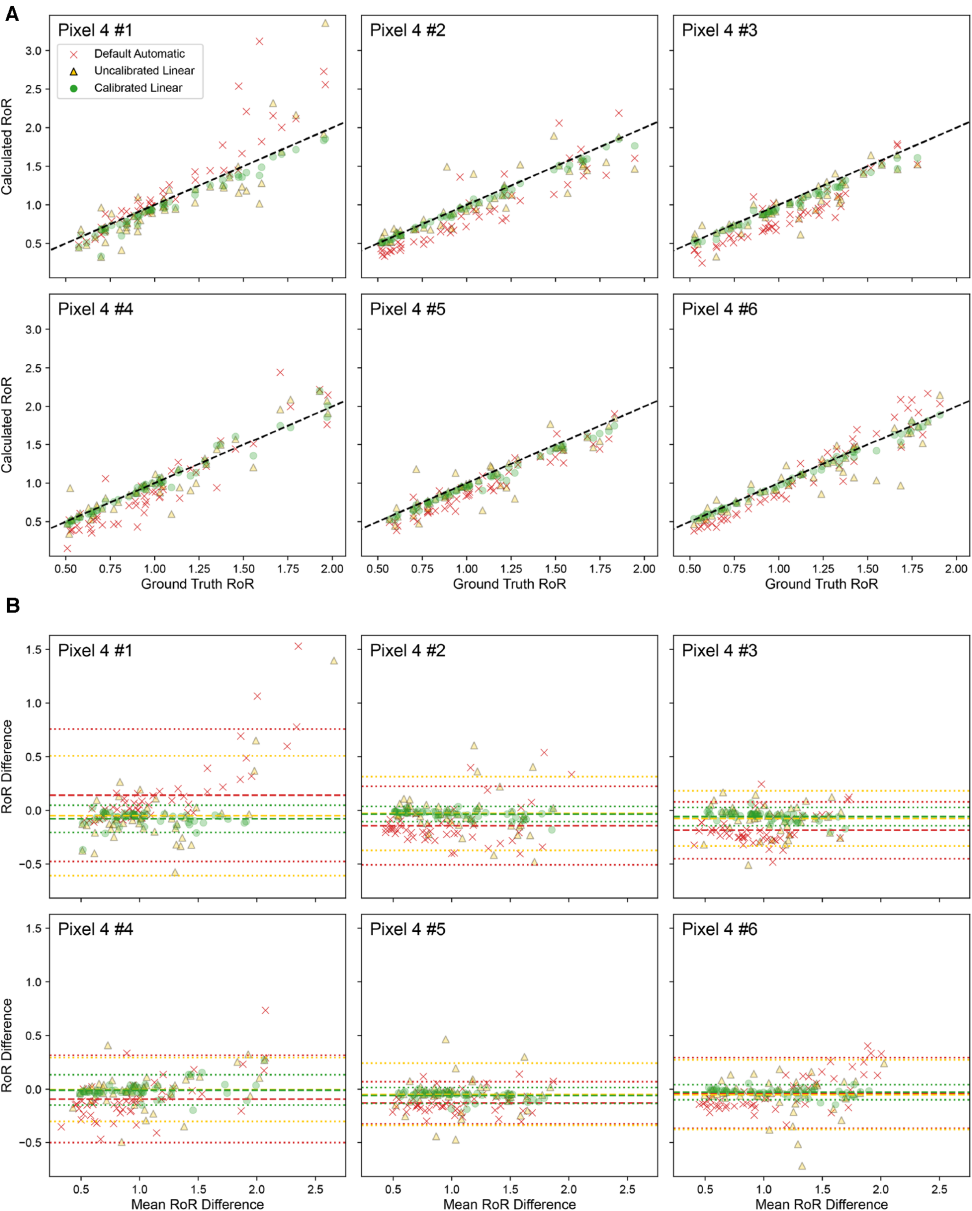
Calibration done for Pixel 4 # 1 is applied to other devices of Pixel 4. (**A**) Correlation plot of ground truth RoR VS Calculated RoR. The dashed line indicates y=x. (**B**) Bland-Altman Plot. The dashed line indicates Bias; the dotted lines indicate limits of agreement (LoA).

The applicability of a calibration performed on one phone to other phones of the same model was further validated. The calibrated value from Pixel 4 #1 was used to calculate the RoR with the recording from five other Pixel 4 devices. As presented in Figure 7, for all six Pixel 4 devices, the Calibrated Linear RoR demonstrated a significantly lower absolute error than both the Uncalibrated Linear RoR and Default Automatic RoR.

In Pixel 4 #1, the Calibrated Linear RoR absolute error was 58% less than the Uncalibrated Linear RoR (p-value=0.009, paired t-test) and 56% less than the Default Automatic RoR (p-value=0.0014, paired t-test). Similar improvements in error reduction were observed across the other Pixel 4 devices. For Pixel 4 #2, the errors were 82% and 67% less ($p\text{-values}=8\times 10^{-15}$ and $1.6\times 10^{-5}$, respectively). For Pixel 4 #3, the errors were 71% and 45% less ($p\text{-values}=2.5\times 10^{-13}$ and $2.5\times 10^{-4}$). For Pixel 4 #4, the errors were 72% and 49% less ($p\text{-values}=7.1\times 10^{-}8$ and $7.1\times 10^{-4}$). For Pixel 4 #5, the errors were 60% and 40% less ($p\text{-values}=4.1\times 10^{-10}$ and $6.7\times 10^{-3}$), and for Pixel 4 #6, the errors were 77% and 69% less ($p\text{-values}=1.9\times 10^{-12}$ and $3.8\times 10^{-5}$). Across devices, on average, the errors were 70% and 55% less ($p\text{-values}=1\times 10^{-4}$ and $1\times 10^{-3}$) (Table 3).

**Table 3:** Calibration performance across six different Pixel 4 devices.

		Pixel 4 device no.	Pixel 4 #1	Pixel 4 #2	Pixel 4 #3	Pixel 4 #4	Pixel 4 #5	Pixel 4 #6	Mean	STD
Correlation	MAE	Default automatic	0.19	0.2	0.21	0.18	0.15	0.15	0.18	0.03
		Uncalibrated linear	0.18	0.11	0.11	0.099	0.1	0.11	0.12	0.03
		Calibrated linear	0.079	0.036	0.061	0.05	0.06	0.034	0.05	0.02
	$R^{2}$	Default automatic	0.86	0.84	0.86	0.88	0.92	0.94	0.88	0.04
		Uncalibrated linear	0.73	0.82	0.83	0.88	0.83	0.84	0.82	0.05
		Calibrated linear	0.97	0.99	0.99	0.97	0.99	0.99	0.98	0.01
Bland-Altman	Bias	Default automatic	0.14	-0.14	−0.18	−0.094	−0.13	−0.037	−0.07	0.12
		Uncalibrated linear	−0.049	−0.029	−0.075	−0.0058	−0.049	−0.053	−0.04	0.02
		Calibrated linear	−0.078	−0.034	−0.058	−0.0095	−0.06	−0.029	−0.04	0.02
	LoA lower	Default automatic	−0.48	−0.51	−0.45	−0.5	−0.33	−0.37	-0.44	0.07
		Uncalibrated linear	−0.61	−0.37	−0.33	−0.31	−0.34	−0.38	−0.39	0.11
		Calibrated linear	−0.2	−0.11	−0.14	−0.15	−0.14	−0.097	−0.14	0.04
	LoA upper	Default automatic	0.76	0.22	0.08	0.31	0.07	0.3	0.29	0.25
		Uncalibrated linear	0.51	0.31	0.18	0.29	0.24	0.27	0.30	0.11
		Calibrated linear	0.047	0.038	0.028	0.13	0.016	0.04	0.05	0.04

The table presents Mean Absolute Error (MAE), $R^{2}$, Bias, Limits of Agreement (LoA Lower and LoA Upper) for each of the three tone mapping settings (Default Automatic, Uncalibrated Linear, and Calibrated Linear). These metrics are used to assess the calibration technique’s effectiveness in improving the accuracy of cPPG measurements across multiple devices of the same model. Mean and standard deviation (STD) values are also included.

These results further underscore the efficacy of our calibration method in enhancing the accuracy of cPPG measurements across multiple devices of the same model, indicating that recalibration for each smartphone of the same model is not necessary.

## 4. Discussion

### 4.1. Recommended camera settings for accurate smartphone cPPG measurements

Based on our experimentation and results, we list the following as a set of recommended settings and parameters that should be followed for accurate and reproducible cPPG measurements.

• Set the tone mapping setting to **Linear**
• Characterize the smartphone camera sensor’s ZLO as outlined in Section 2.2.1 for later use during RoR calculation.
• Disable auto exposure and set fixed ISO and exposure time values to ensure the target signal maintains the desired strength.
• Disable auto white balancing and lock each color channel gain to a fixed value so that the target signals in all color channels are of desired strength. In addition, use an **identity matrix** as the color correction matrix.
• Disable auto focus.
• Perform PPG processing frame by frame in real time to avoid artifacts from video compression algorithms.

It is only possible to manually set the camera tone mapping to linear within the Android Camera 2 API. Such an option is not available in iOS. Although it is possible to capture RAW photos using iOS AVFoundation, the RAW capture mode is not possible for video recordings necessary for cPPG measurements. As such, our current findings and recommended settings are only immediately adaptable to Android devices.

### 4.2. Importance of tone mapping

It’s important to note that the Default Automatic tone mapping setting operates as a black box algorithm, capable of dynamically changing the tone map. Based on our testing, Default Automatic is influenced by the last used tone mapping and adjusts it based on the current scene. Given that most applications would favor the use of high dynamic range, we anticipate the Default Automatic algorithm to employ a nonlinear map that is a shifted version of an sRGB tone map. However, the dynamic nature of the automatic setting, which is used as the default, leads to unpredictable mapping. If not appropriately controlled, this can induce drastic variations in the measurements taken at different instances.

In our experiments, we discovered that tone mapping is a key component in capturing highly linear signals using the smartphone camera. This aspect has been incorrectly configured in all prior work, to the best of our knowledge. When comparing the effect of linear versus nonlinear tone mapping, we observe a notable difference in the RoR calculation, with the LoA being an order of magnitude lower when using a calibrated linear tone map.

### 4.3. Importance of device calibration

Even with the introduction of linear tone mapping, we find that without calibrating the threshold offset for each phone, the recorded PPG signal can still show significant deviation. The DC value (offset due to tissue and diastolic blood volume) is consistently underestimated. This makes sense, as the offset illustrated in Figure 4 where the ZLO leads to a loss in signal but then linearly tracks above it, minimizing the contribution of DC. The effect of the calibration is also prominent in the conversion of the measurement to a ratio measurement. Even though the linear tone mapping results in a lower error than the default automatic setting, it is still an order of magnitude higher than the fully calibrated performance. These findings underscore the importance of performing a calibration on each phone.

We would like to emphasize, however, that the per-phone model calibration is not a burdensome process. As we demonstrated, a calibration made with one instance of a model is applicable to other phones of the same model. This means that each user’s device does not need individual calibration.

Although the need for a physical calibration is not ideal, it is important to note that this form of calibration is biomarker algorithm agnostic. This means that the calibration is only to ensure the measurement from the phone is linear, allowing for faithful recovery of the signal amplitude recorded. This process is distinct from model training typically done for discovering the relationship between pulse measurement and a biomarker (such as SpO2, Hemoglobin, Blood Pressure, etc). The calibration’s goal is to ensure that measurement from one phone is comparable to the measurement from another phone. In this way, when developing a new biomarker to measure with cPPG, the data-driven model trained on data collected on one phone will work on the next, regardless of the phone’s model. Prior work that does not perform such calibration would require recording the human subject data on every phone model that the data-driven model would be used on, which scales poorly. Moreover, given the nature of nonlinear tone mapping, if the exposure or incident light changes, the transformation can be different, leading to unpredictable performance even when used on the same phone model.

Given our findings, we believe that prior work using cPPG for digital biomarker measurements would significantly benefit from the calibration proposed in this paper. Furthermore, this calibration does not need to be conducted by every developer of smartphone cPPG measurement applications. It may be possible to build a shared public lookup table of the calibration value to correct the bias term for each phone model that can be used across applications.

### 4.4. Implications for smartphone medical imaging beyond cPPG

The calibration proposed in this paper suggests that cPPG monitoring in human subjects could achieve 73% higher accuracy compared to default settings. As smartphone health measurements become increasingly prominent, the reliability of the measurement will become increasingly important. Even with the theoretical improvements from our proposed methods, clinical studies with human subjects should be performed to fully understand the accuracy and validity of smartphone clinical measurements.

Beyond cPPG, the influence of default smartphone camera alterations can significantly impact other health measurements. It is likely that remote PPG (rPPG) measurements are similarly influenced by the issues of smartphone-based imaging. More broadly, any image-based medical information collected using smartphones could be influenced. For example, prior work using smartphone cameras to perform jaundice measurement can also be significantly affected by the effects of tone mapping and color correction as the proportionality of colors can be significantly altered depending on the total illumination of the scene. Similarly, techniques for measuring hemoglobin by taking single photos of the nailbed or the inner eyelid may also be significantly affected. In these situations where a single image is needed, we would recommend the use of RAW image capture when available, as this approach eliminates all the issues proposed and is not overly computationally intensive compared to the cPPG scenario where a higher frame rate is needed. When RAW image format is not available, adhering to the guidelines put forth in this paper for maximally linearizing the measurement will provide a close representation.

### 4.5. Limitations

This study relies on the use of benchtop simulations to evaluate our calibration. Although the ideal scenario would involve rigorous testing with human subjects, it is practically unfeasible to test the system in a manner where a range of RoR can be examined without significant physiological intervention. Considering that RoR is associated with variations in blood composition, a comprehensive examination of the wide range of potential RoR values would necessitate manipulating oxygenation or hemoglobin concentration of individuals, all the while measuring them with multiple phones. Although such studies can be conducted with contract research organizations (CRO) as demonstrated in Hoffman et al. (8), these investigations tend to be costly. We advocate that the use of simulation to capture this wide range of RoR serves as a productive initial step in motivating the necessity for system linearization and calibration.

It is worth noting that the same constraints apply when calibrating new designs of pulse oximeters. These devices are regularly calibrated using a similar system that employs an LED and photodiodes to simulate a wide range of RoR, as would be expected from a diverse range of oxygen saturation levels (48). These SpO2 functional testers are a vital component in scaling the manufacturing of systems, as they facilitate device calibration without necessitating repeated human subject testing. In future work, we believe that our calibration solution should also be considered as standard practice for developing smartphone-based pulse monitoring solutions.

The findings from this paper can only be fully adapted to smartphones supporting the Android Camera2API. We identified that the current version of iOS AVFoundation (Xcode 14.3) does not expose the same level of control over tone mapping as Android Camera2API. Without such control, iOS phones will utilize a nonlinear, black box algorithm for tone mapping, which can lead to unreliable PPG measurements. However, it is entirely possible for such control at the firmware level. Also, it is possible that future version of AVFoundation will support such capabilities.

## 5. Conclusion

This paper is the first deep-dive investigation into the nuanced effects of camera parameters on the fidelity of the PPG captured by a smartphone camera. We show a clear dependency on such parameters as tone mapping and color correction settings. Furthermore, we determined that parameter control alone is not enough, and that a per-device model calibration is important. We devised a simple calibration solution using straightforward construction requiring only a dark light shield and a PWM controlled LEDs. We further substantiate our claim through simulation testing across multiple smartphones. The proposed guidelines around the proper use of cPPG can substantially improve the quality of smartphone-based PPG measurements when followed properly, grounding the use of smartphone sensors for producing reliable measurements for a wide array of medical measurements from blood analysis to diabetes screening. This investigation motivates the need for developing a thorough understanding of smartphone-based imaging systems and possible regulation or standardization around smartphone based medical sensing. Incorporating appropriate safeguards in future smartphone-based medical measurements rather than simply relying on data-driven models for correction and feature discovery promotes reliable measurements for the future.

## Data Availability

The raw data supporting the conclusions of this article will be made available by the authors, without undue reservation.
